# Dr. J. Thad. Johnson

**Published:** 1884-12-20

**Authors:** 


### DR. J. THAD. JOHNSON,

Formerly Professor in the SOUTHERN MEDICAL COLLEGE, left Atlanta recently for his new home in the West. Dr. Johnson has been a highly esteemed and eminent member of the profession in Atlanta, and in Georgia. He filled, with ability and distinction, the chair of Anatomy in the Atlanta Medical College, and subsequently was chosen to the chair of Surgery in the Southern Medical College, in which chair he sustained himself with distinction and ability. He bears with him the kind regards and well wishes of his colleagues in the faculty and of numerous citizens and professional friends in this city.

